# SKIP controls lysosome positioning using a composite kinesin-1 heavy and light chain-binding domain

**DOI:** 10.1242/jcs.198267

**Published:** 2017-05-01

**Authors:** Anneri Sanger, Yan Y. Yip, Thomas S. Randall, Stefano Pernigo, Roberto A. Steiner, Mark P. Dodding

**Affiliations:** Randall Division of Cell and Molecular Biophysics, King's College London, London, SE1 1UL, UK

**Keywords:** Kinesin-1, SKIP, Lysosome positioning, Microtubule transport

## Abstract

The molecular interplay between cargo recognition and regulation of the activity of the kinesin-1 microtubule motor is not well understood. Using the lysosome adaptor SKIP (also known as PLEKHM2) as model cargo, we show that the kinesin heavy chains (KHCs), in addition to the kinesin light chains (KLCs), can recognize tryptophan-acidic-binding determinants on the cargo when presented in the context of an extended KHC-interacting domain. Mutational separation of KHC and KLC binding shows that both interactions are important for SKIP–kinesin-1 interaction *in vitro* and that KHC binding is important for lysosome transport *in vivo*. However, in the absence of KLCs, SKIP can only bind to KHC when autoinhibition is relieved, suggesting that the KLCs gate access to the KHCs. We propose a model whereby tryptophan-acidic cargo is first recognized by KLCs, resulting in destabilization of KHC autoinhibition. This primary event then makes accessible a second SKIP-binding site on the KHC C-terminal tail that is adjacent to the autoinhibitory IAK region. Thus, cargo recognition and concurrent activation of kinesin-1 proceed in hierarchical stepwise fashion driven by a dynamic network of inter- and intra-molecular interactions.

## Abstract (precis)

**Summary:** The lysosomal kinesin-1 cargo adaptor SKIP is shown to interact with kinesin-1 via both its heavy and light chains. A new stepwise hierarchical model for kinesin-1 activation is proposed.

## INTRODUCTION

Kinesin-1 (also known as conventional kinesin) is a heterotetrameric microtubule (MT) motor that has diverse roles in both neuronal and non-neuronal cells, where it contributes to control the spatial and temporal organization of many cellular components by virtue of its ability to interact with different cargoes and drive their translocation towards the plus-end of MTs (Fu and Holzbaur, 2014; Vale, 2003; Vale et al.,1985; Verhey and Hammond, 2009b). Kinesin-1 is hijacked by pathogens including vaccinia virus, herpes viruses and HIV-1 during infection, as well as by bacteria that replicate inside the cell such as *Salmonella* (Boucrot et al., 2005; Dodding and Way, 2011; Dumont et al., 2010). It also plays a role in several neurological conditions including Alzheimer's disease and hereditary spastic paraplegia (Bayrakli et al., 2015; Morihara et al., 2014). Understanding how kinesin-1 interacts with its cargos and how it is regulated is important for determining its role in both normal cell function and pathological conditions.

Heterotetrameric kinesin-1 is comprised of two heavy chains (KHCs; encoded by *Kif5A*, *Kif5B* or *Kif5C*), which at their N-terminus contain a motor domain that possesses MT-stimulated ATPase activity, and two light chains (KLCs; encoded by *KLC1–KLC4*), which play an important role in regulation and cargo recognition. An extended series of coiled coils mediate dimerization of the KHCs (Hackney, 2007). The C-terminal region of KHC is largely unstructured. In the absence of cargo binding, kinesin-1 is autoinhibited. This folded, compact state prevents unnecessary cycles of ATP hydrolysis. A C-terminal isoleucine-alanine-lysine (IAK) motif from a single KHC tail binds at the interface between the two motor domains. This acts to crosslink the motor domains, preventing movement of the neck linker region that is required for ADP release (Dietrich et al., 2008; Friedman and Vale, 1999; Hackney and Stock, 2000; Hackney et al., 2009; Kaan et al., 2011; Wong et al., 2009). Cargo binding relieves autoinhibition and results in a more elongated structure that is able to hydrolyze ATP and move along MTs (Blasius et al., 2007; Cai et al., 2007; Friedman and Vale, 1999; Hackney et al., 1992; Kawano et al., 2012).

The KLCs interact with the KHC through a series of heptad repeats (Diefenbach et al., 1998). A highly charged unstructured linker region connects this KHC-binding region to a tetratricopeptide repeat (TPR) domain, which is followed by a variable C-terminal domain that differs between KLC isoforms. As well as binding to cargoes, the KLCs are thought to regulate KHC autoinhibition (Cai et al., 2007; Friedman and Vale, 1999; Fu and Holzbaur, 2014; Verhey et al., 1998; Wong and Rice, 2010). We recently described how a leucine-phenylalanine-proline (LFP) motif in the unstructured KLC linker region engages in a second autoinhibitory interaction, in *cis* with the adjacent TPR domain, to control the interaction of the KLC with a subset of cargoes. This motif acts as a molecular switch to control the conformational state of the light chains and the activity of the motor (Yip et al., 2016).

Vesicular cargoes interact with kinesin-1 via adaptor proteins that can bind to several sites on both KHCs and KLCs (Gindhart, 2006; Hammond et al., 2008; Sun et al., 2011; Watt et al., 2015; Woźniak and Allan, 2006). Cargo recognition by KLCs is accomplished, in part, through the interaction of their TPR domains with short linear peptide ‘W-acidic’ peptide motifs that are found in many cargo adaptors including calsyntenin-1/Alcadein (CSTN1), SifA-kinesin interacting protein (SKIP; also known as PLEKHM2), nesprin-2, gadkin, vaccinia virus A36R, cayman ataxia protein (BNIP-H; also known as ATCAY) and dynein intermediate chain (Aoyama et al., 2009; Araki et al., 2007; Dodding et al., 2011; Kawano et al., 2012; Konecna et al., 2006; Pernigo et al., 2013; Rosa-Ferreira and Munro, 2011; Schmidt et al., 2009; Twelvetrees et al., 2016; Verhey et al., 2001; Wilson and Holzbaur, 2015; Zhu et al., 2012). W-acidic motifs are characterized by a central tryptophan (W) residue that is typically flanked by aspartate (D) or glutamate (E) residues and often preceded by a leucine/methionine (L/M) residue at the W –2 position.

Fusion of these W-acidic motifs to otherwise non-kinesin-1-binding proteins is sufficient to promote kinesin-1-dependent transport or dispersion of specific cellular compartments, such as lysosomes (Dodding et al., 2011; Kawano et al., 2012; Pu et al., 2015). This has also become a useful tool to target cargoes, in a kinesin-1-dependent manner, through the pre-axonal exclusion zone (Farías et al., 2015). Taken together, these studies indicate that KLC binding to W-acidic motifs results in sufficient recruitment and relief of kinesin-1 autoinhibition to allow transport. However, other reports have clearly demonstrated that multiple contacts between cargo and both KHCs and KLCs are important for full motor activity (Blasius et al., 2007; Fu and Holzbaur, 2013, 2014; Verhey and Hammond, 2009a). We therefore sought to examine whether KHC–cargo binding contributes to the capacity of W-acidic motif containing proteins to recruit kinesin-1 and drive cargo transport.

Here, we use the lysosome, melanosome and lytic granule cargo adaptor SKIP, which is mutated in hereditary cardiovascular disease, as a model cargo (Ishida et al., 2015; Muhammad et al., 2015; Rosa-Ferreira and Munro, 2011; Tuli et al., 2013). We show that KHCs, in addition to KLCs, can recognize W-acidic motifs on cargo when presented in the context of an extended interacting domain. In SKIP, both KHC- and KLC-binding sequences are integrated into a kinesin-1-binding cassette. Mutational separation of KHC and KLC binding shows that both interactions are important for SKIP–kinesin-1 interaction *in vitro* and that KHC binding is important for lysosome transport *in vivo*. We propose a model whereby KLCs first recognize the W-acidic motif on cargo, resulting in destabilization of KHC autoinhibition (Kawano et al., 2012; Pernigo et al., 2013; Yip et al., 2016). This makes a second binding site in the C-terminal tail of KHC adjacent to the autoinhibitory IAK region accessible, which then acts to reinforce the motor–cargo interaction and promote efficient transport.

## RESULTS

### The W-acidic motif containing kinesin-1 adaptor protein SKIP, but not CSTN1, interacts directly with the C-terminal cargo-binding tail of KHC

**Fig. 1.**
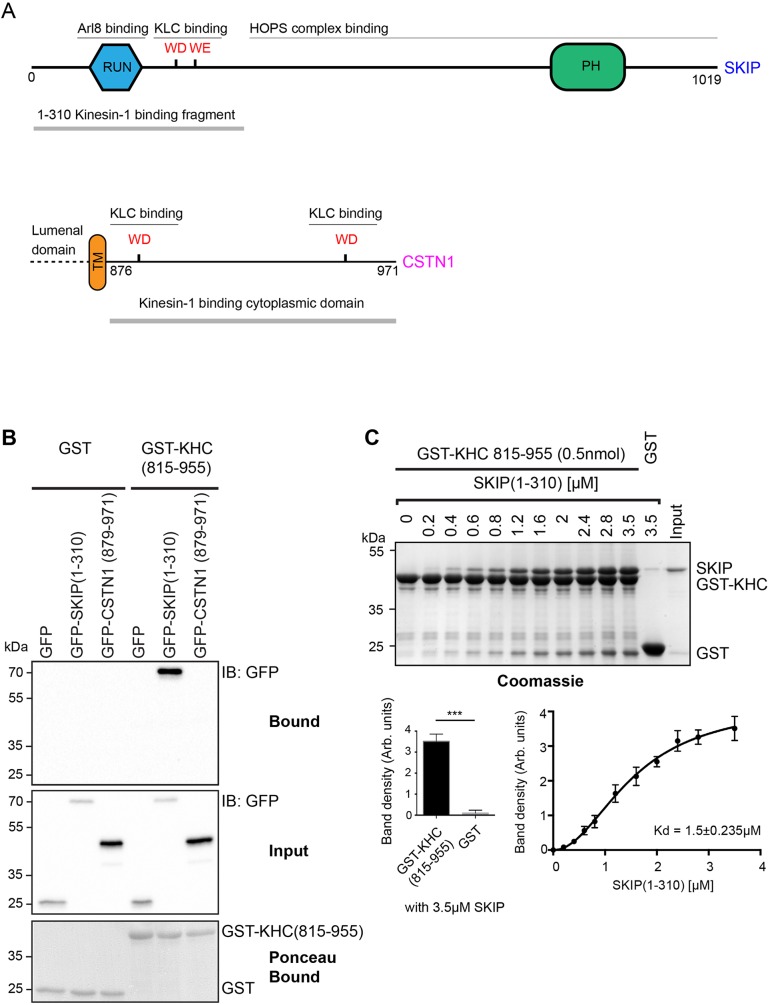
**SKIP interacts directly with KHC.** (A) Schematic showing domain organization of SKIP and CSTN1. (B) Western blot (IB) showing that GST–KHC(815-955) retains GFP–SKIP(1-310) from 293T cell extracts but not the cytoplasmic domain of CSTN1(879-971). Data are representative of three independent experiments. (C) Top: Coomassie-stained SDS-PAGE gel showing results from a GST pulldown experiment showing a direct interaction between GST–KHC(815-955) and SKIP(1-310). Bottom left: graph showing relative binding of SKIP(1-310) to GST and GST–KHC(815-955) at the 3.5 μM concentration shown in the top panel. Data is mean±s.e.m. of three independent experiments. ***P<0.001 (two-tailed *t*-test). Bottom right: graph showing quantification of SKIP(1-310) band density from three independent experiments as shown in the top panel. Error bars show ±s.e.m. Results are fitted to a one-site specific binding model with a hill slope [EQ Y=B_max_×X*^h^*/(*K*_d_^h^+X*^h^*) where B_max_=4.23, *K*_d_=1.53] (*h*)=2.04. R^2^=0.98.

Several studies have indicated that cargo binding to both KHC and KLC may be important to fully activate kinesin-1 and drive efficient plus-end-directed transport (Blasius et al., 2007; Fu and Holzbaur, 2013; Sun et al., 2011; Watt et al., 2015). We tested whether the W-acidic cargo proteins SKIP and CSTN1, which bind to the TPR domain of KLC, could also interact with the cargo-binding C-terminal tail region of KHC. We found that the C-terminal tail of KHC (Kif5C, amino acids 815–955) fused to GST pulled down the N-terminal region of GFP–SKIP(1-310) (which includes its tandem W-acidic motifs) from 293T cell extracts, but not the equivalent cytoplasmic domain of CSTN1(879-971) (Fig. 1A,B). GFP–SKIP was also pulled down by the equivalent region of Kif5B (Fig. S1A).

To test whether SKIP interacts with the KHC directly, SKIP(1-310) was expressed and purified from *E. coli* as a GST fusion protein. The GST moiety was subsequently separated from SKIP(1-310) by cleavage with 3C protease followed by incubation with glutathione beads (Fig. S1B). GST–KHC(815-955) (0.5 nmol) bound to glutathione beads was incubated with increasing concentrations of SKIP(1-310) in solution. GST alone was used as a control at the highest concentration of SKIP(1-310) tested (3.5 μM). Complexes were analysed by SDS-PAGE and Coomassie staining (Fig. 1C, top). Densitometry analysis from three independent experiments suggested that binding approached saturation at the highest concentration of SKIP(1-310) employed, with an apparent 1:1 stoichiometry. Comparison of band density in experiments performed concurrently with 3.5 μM SKIP(1-310) showed that significantly more SKIP was retained by GST–KHC(815-955) than GST alone (Fig. 1C, bottom left). Curve fitting by non-linear regression indicated a dissociation constant (*K*_d_) of approximately 1.5±0.24 μM (mean±s.e.m.; Fig. 1C, bottom right), although this value should be interpreted with caution given the non-equilibrium nature of the experiment, potential for avidity effects and relatively high concentration of the GST–KHC(815-955) receptor. Nonetheless, these data clearly demonstrate that SKIP(1-310) interacts directly with the C-terminal tail of KHC.

### KHC binds SKIP using residues located between amino acids 876 and 917

**Fig. 2.**
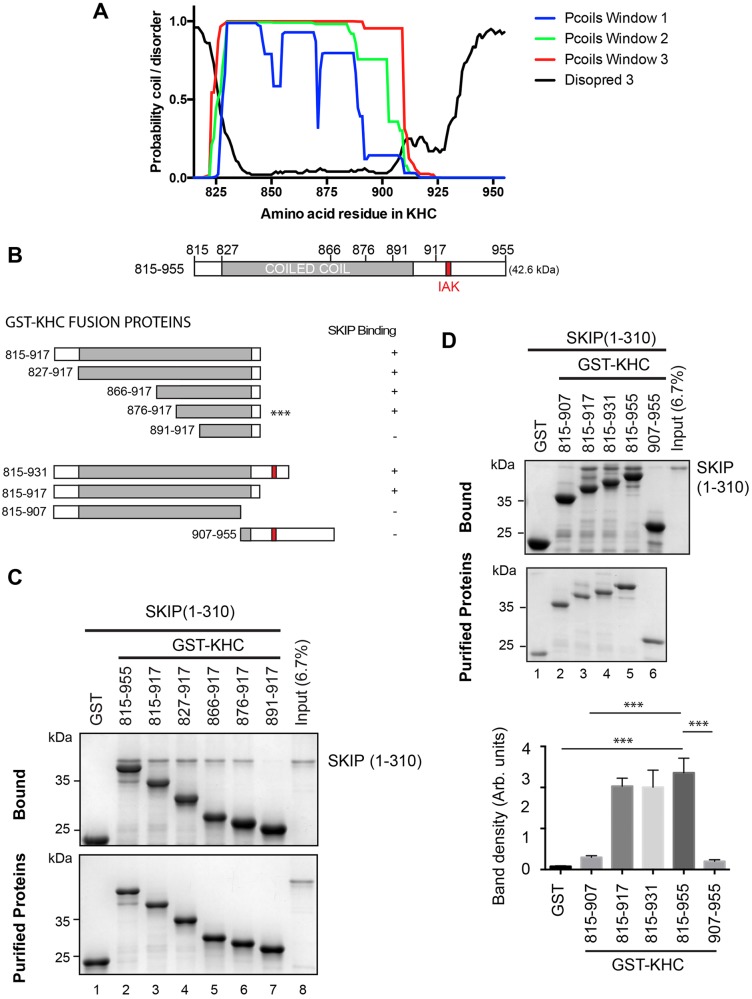
**SKIP–KHC interaction requires amino acids 876–917 of KHC.** (A) Graph and schematic showing structure predictions from Pcoils and Disopred3 for the C-terminal region of KHC. The location of the autoinhibitory IAK motif is highlighted. (B) Schematic of constructs used in this figure with summary of SKIP(1-310)-binding capacity. *** indicates the minimally defined SKIP-binding segment of KHC. (C) Coomassie-stained SDS-PAGE gels from a GST pulldown experiment using a series of truncations of KHC showing that amino acids 876-917 are sufficient for SKIP(1-310) binding. Data are representative of three independent experiments. (D) Coomassie-stained SDS-PAGE gels from a GST pulldown experiment using a series of truncations of KHC showing that amino acids 908–917 are required for SKIP(1-310) binding. Graph shows mean±s.e.m. of relative binding from three independent experiments. ***P<0.001 (one-way ANOVA and Dunnett's multiple comparison test for comparison to 815–955).

Consistent with the studies of Hackney (Hackney, 2007) and Rice (Seeger and Rice, 2013), analysis of the KHC C-terminal region using Pcoils (Lupas et al., 1991) indicated that most of the KHC tail construct is comprised of a coiled coil or series of coils, which terminate between amino acids 900 and 910. Further analysis using Disopred3 (Jones and Cozzetto, 2015) suggests that the most C-terminal region is unstructured (Fig. 2A). We performed GST pulldown experiments using a series of N- and C-terminal truncations of GST–KHC(815-955) (Fig. 2B) to determine where SKIP binds on KHC. Removal of the C-terminal unstructured region (amino acids 918–955), which includes the autoinhibitory IAK motif, did not affect binding to SKIP(1-310) when compared to the longest KHC construct (Fig. 2C, compare lanes 2 and 3). Amino acids 815–875 are also dispensable because a construct comprising residues 876–917 bound SKIP(1-310) to a similar extent to the longest construct (Fig. 2C, compare lanes 2 and 6); however, further N-terminal truncation to 891 dramatically reduced binding (Fig. 2C, compare lanes 6 and 7).

In a separate experiment, a C-terminal truncation of an 815–917 construct to 815–907 also resulted in a reduction in binding (Fig. 2D, compare lanes 2 and 3 and graph columns 2 and 3). Taken together, these data show that the key binding determinants for the KHC-SKIP(1-310) interaction reside within the predominantly coiled coil region between amino acids 876 and 917 on KHC. This segment has previously been implicated in the binding of a number of kinesin-1 cargoes including TRAK2, the Kv3 (Shaw) voltage gated K^+^ channel (also known as KCNC2), fasciculation and elongation protein ζ-1 (Fez1) and HAP1 (Barry et al., 2013; Blasius et al., 2007; Randall et al., 2013; Twelvetrees et al., 2010).

### The KHC-binding site on SKIP overlaps the W-acidic KLC-binding site

**Fig. 3.**
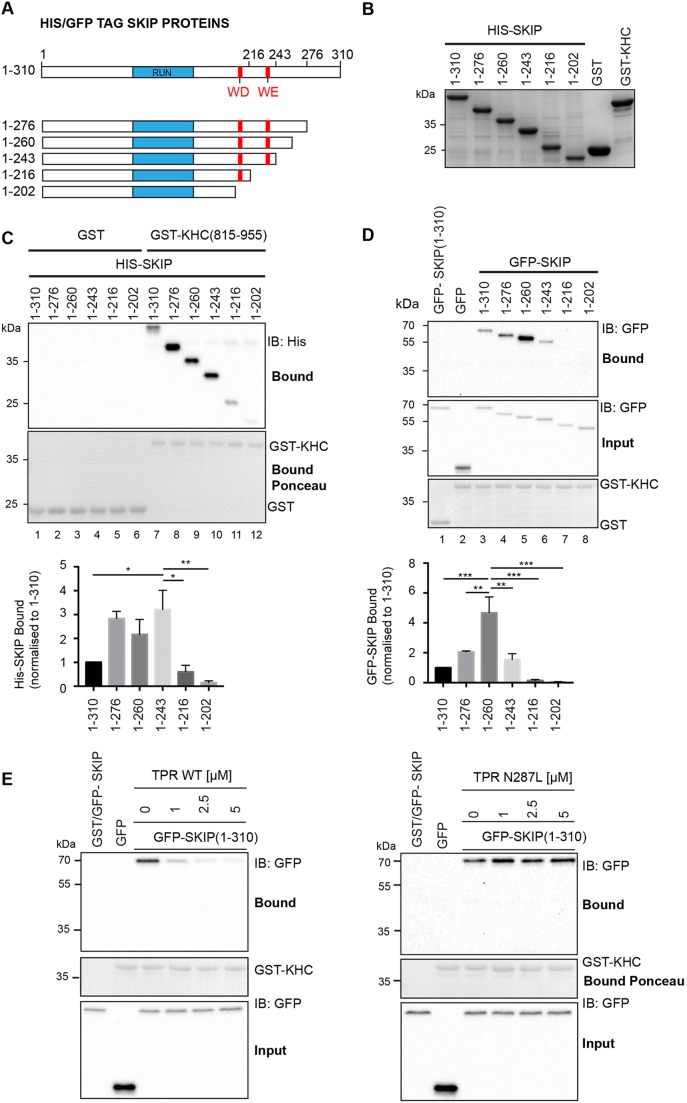
**The KHC-binding site on SKIP overlaps the KLC binding W-acidic motifs.** (A) Schematic of SKIP constructs used in this figure highlighting location of the RUN domain, which binds to Arl8 to recruit SKIP to lysosomes, and W-acidic motifs. (B) Coomassie stained SDS-PAGE gel showing samples of His-tagged SKIP proteins used in C. 20 μl of proteins at 1 μM were loaded. (C) Western blot (IB) showing GST pulldown experiment using GST–KHC(815-955) and various C-terminal SKIP truncations produced in *E. coli.* Binding is reduced when W-acidic motifs are removed. Graphs shows mean±s.e.m. from three independent experiments. *P<0.05; **P<0.01 (one-way ANOVA with Dunnett's multiple comparison test for comparison to 1–243). (D) Western blot showing GST pulldown experiment using GST–KHC(815-955) and various C-terminal SKIP truncations produced in 293T cells. Binding is reduced when W-acidic motifs are removed. Graphs show mean±s.e.m. from three independent experiments. **P<0.01, ***P<0.001 (one-way ANOVA with Dunnett's multiple comparison test for comparison to 1–260). (E) Western blot showing competition experiment where purified recombinant KLC2^TPR^ is added at increasing concentrations to extracts of 293T cells expressing GFP–SKIP variants. Binding to GST–KHC(815-955) is reduced in a concentration-dependent manner by wild-type (WT) KLC2^TPR^ (left) but not KLC2^TPR^ carrying the W-acidic N287L (right) cargo-binding mutation. Data are representative of three independent experiments.

To determine the KHC-binding site on SKIP, SKIP(1-310) and a series of C-terminal truncations (1–276, 1–260, 1–243, 1–216 and 1–202) (Fig. 3A) were expressed in *E. coli* as N-terminally His_6_-tagged proteins and were purified by Ni^2+^-affinity chromatography (Fig. 3B). Pulldown assays, analysed by western blotting with an anti-His_6_-tag antibody, were used to assess the KHC–SKIP interaction. Consistent with our previous experiments indicating a direct interaction between KHC and SKIP, His_6_–SKIP(1-310) was bound by GST–KHC(815-955) but not GST alone (Fig. 3C, compare lanes 1 and 7). We also observed that C-terminal truncation of SKIP to amino acid 276 appeared to enhance binding (Fig. 3C, lane 8). However, binding was dramatically reduced when this construct was further truncated to residue 216 (lane 11), which removes the second W-acidic motif of SKIP (denoted WE). Binding was further reduced by truncation to residue 202, which removes the first W-acidic motif (denoted WD) (lane 12). These data suggest that an extended region of SKIP, encompassing, but not limited to, its W-acidic motifs, contributes to KHC binding. To confirm these findings, the assay was repeated using the same GFP-tagged SKIP proteins expressed in 293T cells. GST–KHC(815-955) did not pull down GFP alone but did efficiently pull down GFP–SKIP(1-310) (Fig. 3D, lanes 2 and 3). Supporting the conclusions from the direct binding experiments, C-terminal truncation of GFP-SKIP(1-310) to residue 260 (lane 5) enhanced the interaction, whereas further truncation from residue 243 (lane 6) to 216 (lane 7) eliminated any detectable interaction in this assay.

The above data imply that the KHC- and KLC-binding sites on SKIP overlap. To test this, we performed a competition experiment where increasing concentrations of purified recombinant KLC2 TPR domain (amino acids 218–480, KLC2^TPR^) were added to 293T cell extracts where GFP–SKIP(1-310) was overexpressed. Binding to KHC was then examined by GST pulldown (Fig. 3E). Addition of KLC2^TPR^ reduced binding to GST–KHC(815-955) in a concentration-dependent manner; Fig. 3E, left). This required a direct interaction between KLC2^TPR^ and SKIP(1-310) because the N287L variant that disrupts the W-acidic SKIP-binding site on KLC2^TPR^ (Pernigo et al., 2013) did not inhibit the SKIP–KHC interaction (Fig. 3E, right).

**Fig. 4.**
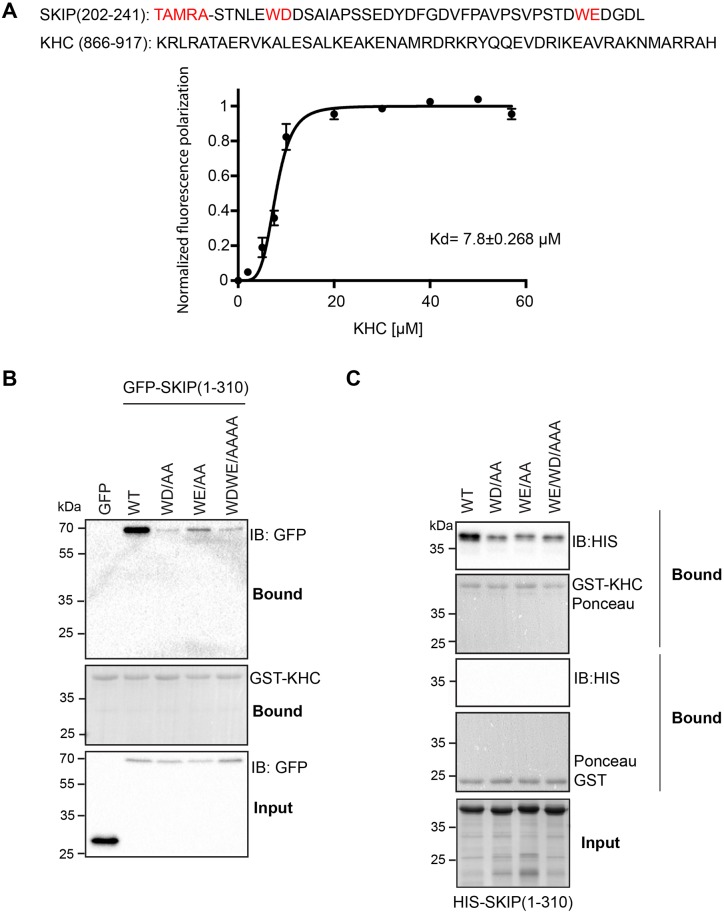
**Interaction between KHC and SKIP requires both W-acidic motifs.** (A) Fluorescence polarization binding assay using a TAMRA-conjugated peptide from SKIP containing both W-acidic motifs. KHC(866-917) was prepared by cleavage of purified GST–KHC(866-917) and removal of free GST or uncleaved protein by incubation with glutathione beads. KHC(866-917) is added at increasing concentration. Error bars show s.e.m. from three replicates and curve shows fit of data to a one-site specific binding model with hill slope [EQ Y=B_max_×X*^h^*/(*K*_d_^h^+X*^h^*) where B_max_=180.7, *K*_d_=7.878] (*h*)=4.703. R^2^=0.98. (B) Western blot (IB) showing GST pulldown experiment from extracts of 293T cells expressing wild-type (WT) GFP–SKIP(1-310) or WD/AA, WE/AA or WDWE/AAAA mutants. Binding is reduced by disruption of either motif. Data are representative of three independent experiments. (C) Western blot showing GST pulldown experiment using purified His_6_–SKIP(1-310) produced in *E. coli.* Binding is reduced by disruption of either W-acidic motif. Data are representative of three independent experiments.

The data described above demonstrate a crucial role for an extended region of SKIP that includes the two W-acidic motifs. We used a fluorescence polarization binding assay to test this directly. Using a TAMRA-conjugated peptide comprising the two W-acidic motifs of SKIP (Pernigo et al., 2013), titration of a KHC construct comprising amino acids 866–917 revealed a concentration dependent and saturable increase in fluorescence polarization (Fig. 4A). The resultant data fit best to a specific binding model with Hill slope (*h*) value of 4 and an equilibrium dissociation constant of 7.8 μM, indicating positive cooperativity. Taken together, these data clearly demonstrate a role for the W-acidic motif-containing KLC-binding segment of SKIP in the KHC–SKIP interaction, but also suggest that residues outside of this region play a role in the interaction. The fact that binding is enhanced by truncation of SKIP(1-310) to residues 276 and 260 may also suggest the presence of a negative regulatory element within this region of the protein.

### SKIP W-acidic motifs are an important determinant for KHC binding

As both W-acidic motifs of SKIP (WD and WE) are present in the minimal KHC-interacting region on SKIP (Fig. 4A), we assessed their contribution to binding directly. Pulldown of GFP–SKIP(1-310) and SKIP mutants (WD/AA, WE/AA, and WDWE/AAAA) from 293T cell lysates by GST–KHC(815-955) showed that binding is dramatically reduced upon making alanine substitutions in either or both of the SKIP W-acidic motifs (Fig. 4B). It is possible that this interpretation may be confounded by endogenous KLC in the cell lysates, although this is unlikely given the typically low expression of KLCs in non-neuronal cells when compared to overexpressed protein. To verify that our results are indicative of a direct effect, we performed equivalent experiments where both proteins were produced in *E. coli.* GST pulldown experiments using His_6_–SKIP(1-310) and SKIP variants WD/AA, WE/AA, and WDWE/AAAA with GST–KHC(815-955) or GST alone again showed that binding is reduced upon making alanine substitutions in the SKIP W-acidic motifs (Fig. 4C). The fact that there is no additive effect from making both substitutions may suggest that both motifs act together in the same binding mechanism.

### Interaction between SKIP and KHC requires relief of KHC autoinhibition

**Fig. 5.**
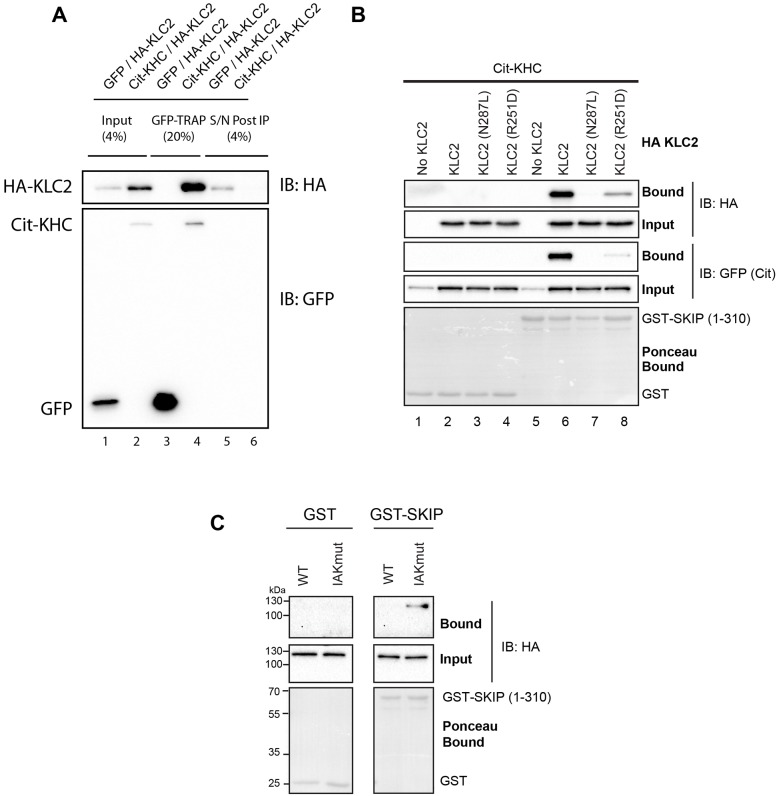
**Interaction between SKIP and full-length KHC requires relief of KHC autoinhibition.** (A) Western blot (IB) of GFP-TRAP immunoprecipitation experiments blot showing that mCit–KHC and HA–KLC2 form a complex when overexpressed in 293T cells. Immunoprecipitation of mCit–KHC results in co-depletion of HA–KLC2 from cell extracts. (B) Western blot showing results of GST–SKIP(1-310) pull down from transfected 293T cells expressing mCit-tagged KHC with or without KLC2 (wild-type or W-acidic cargo-binding mutants N287L and R251D). The ability to pulldown the kinesin-1 tetramer is disrupted by W-acidic-binding mutations in KLC2. KHC expressed alone is not retained by GST–SKIP(1-310). Data are representative of three independent experiments. (C) Western blot showing results of GST–SKIP(1-310) pulldown from transfected 293T cells expressing HA–tagged KHC, either wild type (WT) or with the autoinhibitory IAK sequence mutated (IAKmut, QIAKPIRPG/AAAAAAAA). Binding to GST–SKIP(1-310) is only detected when autoinhibition is relieved. Data are representative of three independent experiments.

Having established some key binding determinants on both KHC and SKIP, we next examined the interaction of SKIP with full-length KHC and the kinesin-1 complex. To confirm that overexpressed KHC and KLC form a complex, 293T cells were transfected with KHC N-terminally tagged with citrulline (mCit, a GFP derivative) (Cai et al., 2007) and HA–KLC2, or, as a control, GFP and HA–KLC2. Co-transfection of mCit–KHC with HA–KLC2 resulted in an increase in HA–KLC2 expression compared to that seen upon co-transfection with GFP (Fig. 5A, compare lanes 1 and 2). mCit–KHC was bound (lane 4) and efficiently depleted from cell extracts (Fig. 5A, compare lanes 2 and 6) using GFP-TRAP beads. HA–KLC2 co-immunoprecipitated with mCit–KHC, but not GFP (Fig. 5A, lanes 3 and 4), resulting in its total depletion from the cell extracts (compare lanes 2 and 6). Taken together, these data show that essentially all of mCit–KHC and HA–KLC2 form a complex with each other. Next, GST-tagged SKIP(1-310) was purified from *E. coli* and bound to glutathione beads. GST–SKIP(1-310) beads (or control GST beads) were incubated with 293T cell extracts expressing N-terminally mCit-tagged KHC (Blasius et al., 2007) alone or mCit–KHC coexpressed with either wild-type or the N287L/R251D mutants of HA–KLC2 that disrupt binding to SKIP (Pernigo et al., 2013). As expected, when co-expressed, both HA–KLC2 and mCit–KHC were pulled down efficiently by GST–SKIP(1-310) (Fig. 5B, lane 6), but not by GST alone (Fig. 5B, lane 2), from extracts expressing wild-type KLC2. Consistent with results shown in Fig. 5A, co-expression of KLC with KHC also results in an increase in KHC expression (Fig. 5B, lanes 5 and 6). Disruption of the W-acidic binding site on KLC using the N287L/R251D mutations dramatically reduced binding of the kinesin-1 complex KLCs, confirming that KLC interactions are important for SKIP–kinesin-1 interaction (Fig. 5B, lanes 7 and 8). Surprisingly, however, we were unable to detect an interaction between GST-SKIP(1-310) and KHC in the absence of KLC (Fig. 5B, lane 5). We reasoned that this may be caused by KHC autoinhibition limiting access to the SKIP-binding site that lies adjacent to the KHC IAK motif. To test this hypothesis, nine residues of the autoinhibitory IAK sequence were mutated to alanine (QIAKPIRPG/AAAAAAAA) to disrupt the binding of the C-terminal tail to the motor domain of KHC and prevent autoinhibition (Dietrich et al., 2008; Hackney and Stock, 2000). HA–KHC constructs were expressed in 293T cells and binding to SKIP(1-310) analysed by GST pulldown (Fig. 5B). When the IAK sequence is disrupted, GST–SKIP(1-310) bound to full-length KHC, suggesting that interaction with SKIP requires relief of KHC autoinhibition.

### Specific disruption of SKIP–KHC binding reduces its capacity to associate with the kinesin-1 tetramer

**Fig. 6.**
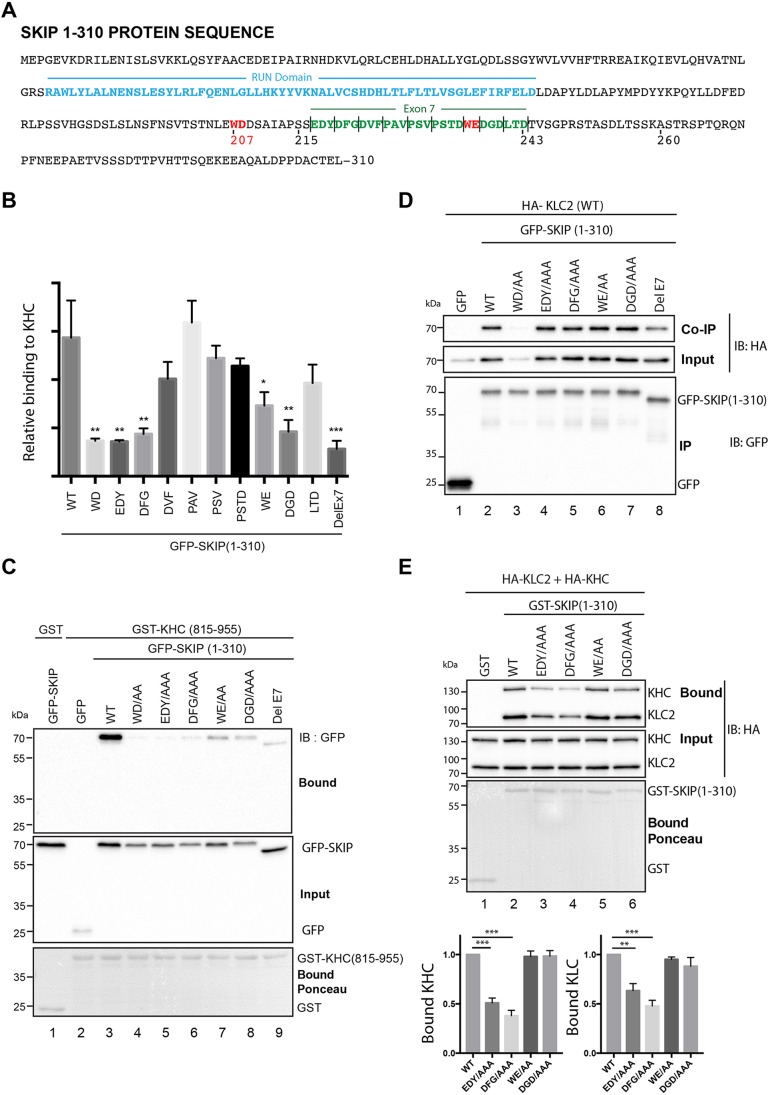
**Specific mutations in SKIP can separate KLC and KHC binding capacity.** (A) Amino acid sequence of SKIP(1-310). The RUN domain as predicted by EMBL S.M.A.R.T. is highlighted in blue. Exon 7 is shown in green. WD and WE motifs are in red. Clusters of amino acid mutations in exon 7 are highlighted by the ‘|’ symbol. (B) Graph showing quantification of relative binding to GST–KHC(815-955) by from extracts of 293T cells expressing wild-type (WT) GFP–SKIP(1-310) and the indicated alanine mutants. Data are from three independent experiments, and were normalized to input levels of GFP–SKIP proteins. Error bars show±s.e.m. *P<0.05, **P<0.01, ***P<0.001 (one-way ANOVA and statistical significance of comparisons to WT using Dunnett's multiple comparison test). (C) GST–KHC(815-955) pulldown experiment with GFP–SKIP(1-310) showing effect of the indicated mutations on the SKIP–KHC interaction. IB, immunoblot. (D) GFP-TRAP immunoprecipitation experiment from extracts of 293T cells transfected with GFP–SKIP(1-310) carrying the indicated mutations and HA–KLC2. KLC binding is not perturbed by EDY, DFG, WE and DFG mutations. Data are representative of three independent experiments. (E) GST-SKIP(1-310) pulldown using wild-type and KHC-binding mutants from transfected 293T cells expressing HA-tagged KHC and HA–KLC2. Binding to the tetramer is reduced by EDY/AAA and DFG/AAA mutations. Graph shows the mean±s.e.m. of binding from three independent experiments. **P<0.01, ***P<0.001 (one-way ANOVA and Dunnett's multiple comparison test for comparison to WT).

The data described above show that amino acid residues 202–241 of SKIP contains both KLC- and KHC-binding sites, although they also suggest an additional role in KHC binding for sequences outside of this region. Moreover, KLC and KHC interactions share some determinants because mutation of the first W-acidic motif (WD/AA) in SKIP not only disrupts KLC binding but also inhibits SKIP binding to KHC. However, mutation of the second (WE) motif in SKIP (WE/AA) has no effect on KLC binding (Pernigo et al., 2013) but does disrupt the interaction with KHC. This suggests that other residues within this region may also be differentially required for KLC and KHC binding. Interestingly, a naturally occurring splice variant of SKIP, where exon 7 is deleted, results in removal of residues 219–238 (highlighted in green in Fig. 6A) (Rosa-Ferreira and Munro, 2011). This splice variant has a reduced capacity to induce lysosome dispersion (Rosa-Ferreira and Munro, 2011) suggesting that this effect could, in part, be due to its reduced KHC binding capacity. We therefore sought to identify mutations in SKIP that reduced KHC but not KLC binding by using an alanine-scanning approach targeting 3- or 4-amino-acid clusters within exon 7 of SKIP. 293T cells were transfected with plasmids designed to express green fluorescent protein (GFP)-tagged SKIP, or SKIP alanine mutants [WD (control for KHC and KLC), EDY, DFG, DVF, PAV, PSV, PSTD, WE, DGD, LTD] or the splice variant with exon 7 deleted (ΔEx7). The results from this screen showing relative binding to GST-KHC are summarized in Fig. 6B and indicated a particularly important role for the EDY(217-219) (Fig. 6C, lane 5), DFG(220-222) (lane 6) and DGD(238-240) (lane 8) triplets in addition to the WD and WE motifs (lane 4 and lane 7). Removal of the whole of exon 7 also reduced binding (Fig. 6C, lane 9).

To determine whether the amino acid replacements that disrupt SKIP binding to KHC were also important in KLC binding, we performed a GFP-TRAP immunoprecipitation experiment. 293T cells were co-transfected with plasmids designed to express HA-tagged KLC2 and GFP-SKIP(1-310) (either wild type, or carrying alanine substitution at WD, EDY, DFG, WE and DGD or with exon 7 deleted). GFP-SKIP(1-310) was immunoprecipitated with GFP-TRAP beads and the amount of co-immunoprecipated HA-KLC2 was analysed by western blot. Co-expression of GFP-SKIP(1-310) with HA-KLC2 (in the absence of KHC) results in stabilization of HA-KLC2 and an increase in relative expression compared to GFP/HA-KLC2 transfected cells (Pernigo et al., 2013). As expected, the WD/AA replacement eliminated binding and suppressed this increase in expression, whereas the WE mutation had no effect (Pernigo et al., 2013) (Fig. 6D, lanes 3 and 6). Deletion of exon 7, which encompasses the WE motif and surrounding residues also slightly reduced binding, suggesting that these residues do make some additional contribution to KLC2 binding (Fig. 6D, lane 8). However, AAA replacement of EDY, DFG, or DGD triplets (Fig. 5D, lanes 4-6) had no effect on expression or the interaction between SKIP and KLC2, confirming that KLC and KHC binding capacity in SKIP is separable.

To determine the effect of independently disrupting SKIP:KHC binding on association of SKIP with the kinesin-1 tetramer, 293T cells were co-transfected with plasmids to express HA-KLC2 and HA-KHC. Pull-down of HA-KLC and HA-KHC from 293T cell lysates using GST-SKIP(1-310) or GST-SKIP(1-310) carrying alanine substitutions in KHC binding residues (EDY, DFG, WE, DGD) confirmed a key role for the EDY or DFG residues (Fig. 6E, lanes 3 and 4), although the mutation the WE or DGD residues had little obvious effect on binding in this assay.

To determine whether mutations in SKIP that disrupt interactions with the kinesin-1 tetramer also affect its ability to promote the association of kinesin-1 with MTs, HeLa cells were transfected with GFP, GFP-SKIP (1-310) or GFP-SKIP(1-310) carrying alanine substitutions in WD, WE, EDY or DFG residues. Cells were lysed in a MT stabilizing buffer and subjected to ultracentrifugation to pellet intact MTs. Consistent with a capacity to promote kinesin-1-MT association, expression of GFP-SKIP promoted an increase in the amount of KHC and KLC in the MT fraction (Fig. S2) (compare left blot lanes 2 and 4 and right blot lanes 10 and 12). KHC and KLC in the pellet was reduced by the WD/AA, WE/AA, EDY/AAA and DFG/AAA mutations in SKIP (lanes 6, 8, 14, 16).

Collectively, these experiments show that the capacity for SKIP to interact specifically with KHC is important for maintaining a stable interaction with kinesin-1 tetramer under conditions where KLC binding is not perturbed.

### Addition of SKIP–KHC specific binding determinants to the C-terminus of CSTN1 promotes KHC binding in a CSTN WD motif-dependent manner

Next, we sought to test directly whether the KHC-binding determinants we had identified in the region of SKIP following its WD motif were sufficient to impart KHC-binding capacity on CSTN1, which does not bind KHC. Taking advantage of the fact that the second WD motif of CSTN1 is positioned close to its C-terminus, a series of CSTN1–SKIP chimeras were engineered that incorporate the cytoplasmic domain of CSTN1 fused N-terminally to GFP, followed by KHC-binding region of SKIP beginning at S215 (thus excluding the SKIP WD motif) (Fig. S3). This places the second WD motif of CSTN1 in a similar relative sequence position. The chimeras terminated at either residue 241 in SKIP [named GFP–CSTN1–SKIP(short)] or residue 260 in SKIP [named GFP–CSTN1–SKIP(long)]. The GFP fusion proteins were expressed in 293T cells and their ability to bind to GST–KHC(815-955) was assessed by pulldown followed by western blotting. As expected, GFP–SKIP (Fig. S3B, lane 2) but neither GFP (lane 1) or GFP–CSTN1 (lane 3) bound to GST–KHC; however, inclusion of amino acids 215–241 of SKIP resulted in the retention of GFP–CSTN1–SKIP(short) (lane 4) and [consistent with our truncation experiments (Fig. 3D)] binding was further enhanced by extension of the SKIP component of the chimera to residue 260 (Fig. S3B, lane 5). As expected, mutation of the KHC-binding EDYDFG cluster in SKIP prevented the interaction (Fig. S3B, lane 6). In addition, the interaction was also disrupted by mutation of the second WD motif of CSTN1 (Fig. S3B, lane 7). Thus, the second WD motif of CSTN1 is functionalized as a KHC-binding determinant by being placed in the context of additional specific SKIP–KHC binding determinants.

### Specific disruption of SKIP–KHC binding capacity reduces its ability to promote the dispersion of lysosomes

**Fig. 7.**
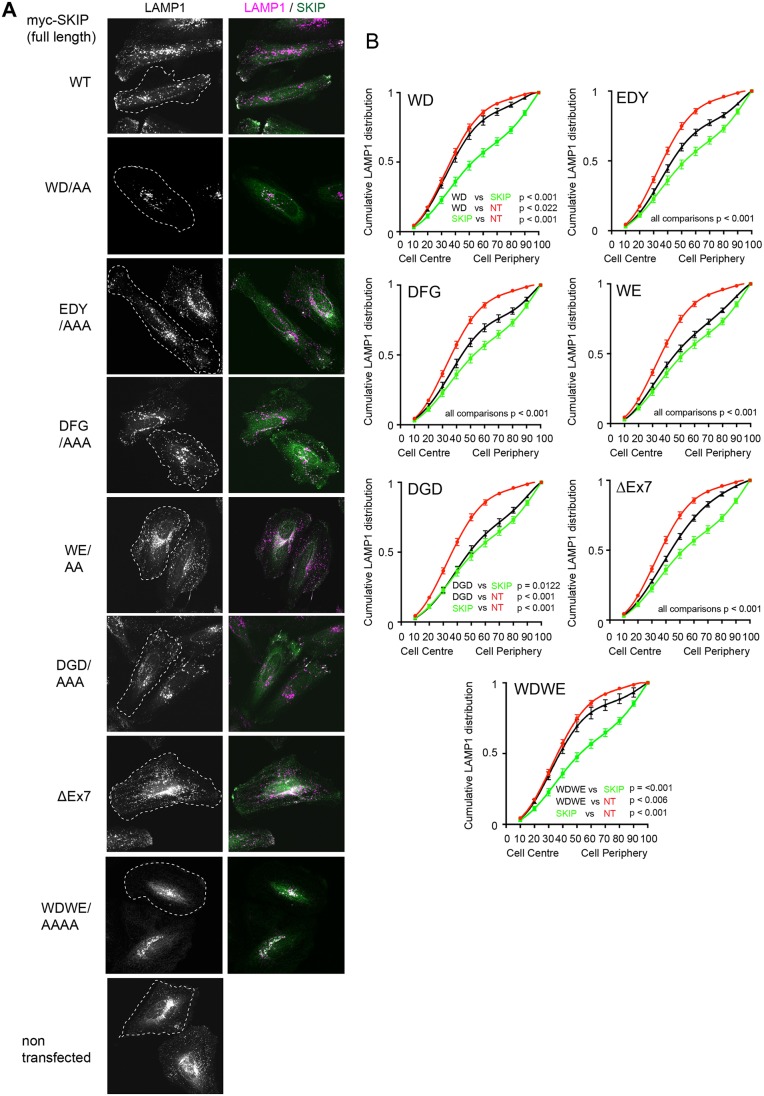
**Specific disruption of SKIP–KHC binding limits the capacity of SKIP to promote the peripheral dispersion of lysosomes.** (A) Immunofluoresence images showing late endosomes and lysosomes (LAMP1, magenta) in HeLa cells transfected with full length Myc–SKIP (green) and the indicated KHC-binding mutants. (B) Graphs showing quantification of lysosome distribution from 45 cells in 3 replicates. Error bars show ±s.e.m. Non-transfected (NT, red) and wild-type Myc–SKIP-transfected (green) curves are reproduced across all graphs. Black line indicates the distribution of lysosomes in cells transfected with the indicated mutant. Statistical analysis of pairwise comparison of curves was carried out as described in the Materials and Methods.

Identification of the residues that specifically disrupted SKIP binding to KHC offered an opportunity to determine their functional role in kinesin-1-dependent transport. Expression of full-length SKIP in HeLa cells promotes kinesin-1 recruitment to lysosomes and their translocation to the cell periphery (Fig. 7A) (Rosa-Ferreira and Munro, 2011). This provides a useful assay to assess kinesin-1-dependent SKIP function. As the intracellular distribution of lysosomes does vary from cell to cell, we used an unbiased quantitative approach to assess the extent of this effect. The cell is segmented into scaled percentile regions and the cumulative LAMP1 distribution from the inner to peripheral regions is measured. A shift of the curve to the right indicates relative dispersion. Curve fitting and analysis using the extra sum of F-squares test allows statistical comparison of the datasets (Fig. 7B) (Starling et al., 2016). As expected, mutation of the SKIP WD motif (which reduces both KHC and KLC binding), or mutation of the WD and WE motif together, essentially eliminated the capacity of SKIP to promote the dispersion of lysosomes (Fig. 7A) (Rosa-Ferreira and Munro, 2011). Representative confocal fluorescence images of the lysosomal distribution are shown in Fig. 7A. To determine the effect of SKIP mutations shown to disrupt KHC, but not KLC, binding to SKIP on the distribution of lysosomes, HeLa cells were transfected with Myc–SKIP, SKIP triplet AAA variants (EDY, DFG, WE, DGD) or the ΔEx7 splice variant. Transfection with the KHC specific EDY, DFG or DGD to AAA mutations significantly reduced their capacity to promote lysosomal dispersion in a manner that correlated well with their capacity to interact with the kinesin-1 tetramer (Fig. 7A,B and Fig. 6E) demonstrating a key role for KHC binding in SKIP-induced late endosome/lysosome dispersion.

## DISCUSSION

Despite its key role in wide range of cellular processes and human diseases, the molecular mechanisms that allow kinesin-1 to recognize its cargoes, and how cargo binding then subsequently regulates kinesin-1 activity are not well understood. Here, using SKIP as a model cargo adaptor, we have further explored these mechanisms and examined how they are coupled to drive efficient transport along MTs. We have shown that KHCs, in addition to KLCs, can recognize W-acidic-based determinants on cargo, but only when presented in the context of an extended interacting sequence. In SKIP, both KHC- and KLC-binding determinants are integrated into a kinesin-1-binding cassette. Mutational separation of KHC and KLC binding shows that both interactions are important for SKIP–kinesin-1 interaction *in vitro* and KHC binding contributes to lysosome transport *in vivo*. Interaction between full-length KHC and SKIP does not occur without the relief of KHC autoinhibition. Taking this analysis together with our previous work and that of others, we propose that the KLCs first recognize W-acidic cargo, which in turns causes a conformational change in KLC (Yip et al., 2016) that results in destabilization of KHC autoinhibition (Kawano et al., 2012; Yip et al., 2016). This makes the second SKIP-binding site in the C-terminal tail of KHC that is adjacent to the autoinhibitory IAK region accessible. Binding of SKIP to this secondary KHC site then acts to reinforce the motor–cargo interaction and maintain KHC in its active state, hence, promoting efficient transport (Fig. S4).

Although the cargo-binding tail of KHC and the TPR domains of KLC share no sequence similarity or structural homology, they can both recognize the same determinants on cargo. Our previous work demonstrated that the KLC^TPR^ interaction with W-acidic motifs is driven by sequence-specific interactions with hydrophobic leucine and aromatic tryptophan residues combined with a large electrostatic component, where negatively charged aspartate and glutamate residues within the W-acidic motif make a network of hydrogen bond and salt bridge contacts with arginine and lysine residues on the concave groove of the TPR domain (Pernigo et al., 2013). In this context, it is interesting to note that a minimal interacting cassette for SKIP with KHC or KLC lacks any predicted secondary structure but is also highly negatively charged with a predicted isoelectric point (pI) of 3. A similar analysis of the minimal SKIP-binding region of KHC(876-917) reveals it to be highly positively charged with a predicted isoelectric point higher than 10. Thus, it seems likely that electrostatic interactions will also play an important role in KHC–cargo recognition. Consistent with this notion, the minimally defined KHC-binding regions of three other cargoes that interact with this region of KHC, namely, TRAK2, Kv3.1 T1 and HAP1 (Barry et al., 2013; Randall et al., 2013; Twelvetrees et al., 2010), also have a low predicted isoelectric points of ∼4.3, 4.5 and 4.2, respectively, and in the case of Kv3.1 T1, a basic RKR triplet in KHC(892-894) has been shown to be essential for binding (Barry et al., 2013). It is interesting to note that Kv3.1 T1, but not the equivalent domains of related ion channels that do not bind to KHC (Xu et al., 2010), contains a highly electronegative and unstructured region (as predicted by Disopred3; sequence DSFGGAPLDNSADDADADGPGDSGDGEDELEMTKRLALSDS, pI 3.25). Similarly, HAP1 contains a negatively charged unstructured region (sequence DSDDDDDEEDEEDEEEGEEEEREGQRDQDQQHDHPYGAPK, pI 3.72) that resides between the first and second coiled coils within its minimal KHC-binding domain (Twelvetrees et al., 2010). Moreover, kinesin-1 can bind and transport negatively charged beads in the squid giant axon (Seamster et al., 2012; Terasaki et al., 1995). However, amino acid sequence, in addition to charge, can also make a contribution in this system because beads coated with a C-terminal peptide from amyloid precursor protein (sequence GYENPTYKFFEQMQN, pI 4.5) were transported more efficiently than beads coated with the same sequence when scrambled (Seamster et al., 2012).

The data presented here show that whereas SKIP interacts with KHC, another W-acidic motif-containing protein, CSTN1 does not. Neither does CSTN1 posses a motif resembling the EDYDFG cluster found in SKIP that is important for KHC binding. Thus, KHC binding is not a universal feature of W-acidic motif-containing proteins, but instead depends upon the sequence context in which W-acidic motifs are presented. This notion is supported by the fact that the second CSTN1 WD motif becomes a determinant for KHC interaction, when fused to SKIP (Fig. S3). Our competition experiments strongly suggest that SKIP cannot bind both KHC and KLC simultaneously (Fig. 3E). Two possible models present themselves. In both, SKIP first interacts with the TPR domain of KLC, which results in a conformational change within the light chains that destabilizes KHC autoinhibition (Kawano et al., 2012; Yip et al., 2016). This makes the second SKIP-binding site on KHC accessible. KLC essentially gates access to KHC. One could then envisage a transfer of cargo from KLC to KHC, as both binding affinities are in the low micromolar range, or perhaps dynamic exchange of SKIP between the two sites. Alternatively, additional SKIP molecules associated with the surface of the lysosome via Arl8b may bind the newly exposed site. Multiple contacts with the kinesin-1 tetramer, would enhance avidity and thus would be predicted to result in a highly stable motor–cargo interaction, such as those found on cellular prion protein (PrPC)-containing vesicles (Encalada et al., 2011). In the context of CSTN1, a secondary set of KHC contacts could be provided by another adaptor associated with the same vesicles. Alternatively, the presence of two relatively high-affinity KLC-binding WD motifs in CSTN1 (Araki et al., 2007; Konecna et al., 2006; Pernigo et al., 2013) may act to circumvent the requirement for KHC binding. Indeed, fusion of multiple, sequential WD motifs to Nipah Virus F glycoprotein (NiV-F) resulted in a greater capacity to transport cargoes into axons when compared to single motifs (Farías et al., 2015).

Within the context of SKIP function in the transport of lysosomes, melanosomes and lytic granules (Ishida et al., 2015; Rosa-Ferreira and Munro, 2011; Tuli et al., 2013), it is interesting to note that some important KHC-binding determinants reside within exon 7. Whereas full-length and ΔEx7 forms of SKIP are expressed in a wide variety of tissues, their relative expression varies considerably (Rosa-Ferreira and Munro, 2011). Therefore, it could be that the transport activity of SKIP is controlled by altering its capacity to interact with KHC and that the quite heterogeneous steady-state distribution of lysosomes found when comparing different cell types is in part be regulated by this. This would allow cells to tightly control this aspect of SKIP function presumably without perturbing other interactions, for example, with the HOPS complex (Khatter et al., 2015). It would also seem worth investigating whether the relative proportion of these two splice variants is regulated more acutely by signaling pathways that control lysosome- and lysosome-related organelle transport. W-acidic motifs are also found in neuronal-specific splice variants of dynein intermediate chain, where kinesin-1 binding is required for its slow transport down the axon (Twelvetrees et al., 2016) suggesting that alternative splicing of key kinesin-1-binding determinants has a more general application for regulation of cargo attachment. It is tempting to speculate that this inherently modular kinesin-1-binding capacity (Dodding et al., 2011), in the form of an unstructured protein region, may have evolutionary implications for the acquisition of the capacity of organelles to move on MTs. Alternatively, it also seems possible that such sequences could be generated by mutations that generate aromatic and hydrophobic amino acids in preexisting negatively charged unstructured regions of proteins.

In summary, we have shown that both KLC and KHC can recognize very closely related determinants on cargo that, in the case of SKIP, are incorporated into a KLC- and KHC-binding cassette. Both interactions contribute to efficient motor recruitment. These data suggest some shared general principles for both heavy and light chain cargo recognition, as both involve a significant electrostatic component combined with shared sequence specific elements, and indicate that motor–cargo attachment is a dynamic, multi-step process.

## MATERIALS AND METHODS

### Plasmids and cells

Rat kinesin-1 heavy chain (KHC, Kif5C) was obtained as an N-terminally mCit-tagged clone (Cai et al., 2007). The full length of Kif5C was amplified by PCR and subcloned into CB6-HA to allow it to be expressed with an N-terminal HA epitope tag. Shorter C-terminal tail fragments were amplified by PCR and subcloned into pMW-GST for expression in *E. coli*. GST vectors contained a 3C protease cleavage site between GST and target protein to allow removal of the affinity tag when required. Myc–SKIP full length and ΔEx7 constructs were a gift from Sean Munro, MRC-LMB, Cambridge, UK. SKIP(1-310) and the indicated truncated variants were amplified from those plasmids and subcloned into pMW-GST, His_6_-(pET28-His) or CB6-GFP as required for expression in bacterial (His and GST) and mammalian (GFP) systems. GFP–CSTN(879–971), HA–KLC2, His–KLC2^TPR^ (218–480) and His–KLC2^TPR^ (218–480) with the N287L mutation were described previously (Pernigo et al., 2013). Human Kif5B was obtained from addgene (plasmid ID 15284, deposited by Ron Vale; Navone et al., 1992) and the C-terminal tail fragment (814–963) was amplified by PCR and subcloned into pMW-GST. For expression of SKIP–CSTN chimeric proteins, DNAs encoding the indicated protein sequences were synthesized by Genscript (NJ, USA) and subcloned into CB6-GFP. All mutations were introduced by site-directed mutagenesis and all plasmids were verified by DNA sequencing. HeLa and 293T cells used in this study were validated by the Cancer Research UK cell services, London Research Institute, and were regularly tested for contamination.

### Protein expression and purification

Proteins were expressed in *Escherichia coli* BL21(DE3) cells. Briefly, single colonies were picked and grown at 37°C overnight. Small-scale overnight bacterial cultures were used to inoculate four 1 l cultures that were incubated at 37°C until they reached an optical density at 600 nm (OD600) of 0.5. The temperature was then lowered to 16°C, and protein synthesis was induced by the addition of 300 μM isopropyl β-D-1-thiogalactopyranoside for 16 h. Cells were harvested by centrifugation at 5000 ***g*** for 15 min at 4°C. For GST-tagged proteins, cells were resuspended in lysis buffer (25 mM HEPES pH 7.5, 500 mM NaCl, 5 mM β-mercaptoethanol) supplemented with protease inhibitor mixture (Roche). Lysis buffer containing 20 mM imidazole was used to purify His-tagged proteins. Cell lysis was accomplished by sonication. Insoluble material was sedimented by centrifugation at 16,500 ***g*** for 30 min at 4°C. GST-tagged proteins were obtained via batch purification using glutathione–Sepharose beads (GE Life Sciences), and His-tagged proteins were purified using His-trap FF columns (GE Life Sciences). Purified proteins from both methods were dialyzed overnight against glutathione or imidazole-free lysis buffer, respectively. His-tagged protein samples were further purified by size-exclusion chromatography (SEC) on a 16/60 HiLoad Superdex 75 column (GE Healthcare). As indicated, the GST tag on fusion proteins was removed by incubating overnight at 4°C using 3C Protease (GE Healthcare), according to the manufacturer's protocol. This was followed by a second 4 h incubation with glutathione beads to remove free GST, 3C protease and any residual uncleaved protein.

### Immunoprecipitation and GST pulldowns from cell extracts

Cells were harvested in 1 ml lysis buffer [25 mM HEPES pH 7.5, 150 mM NaCl, 0.1% NP-40, 0.1× Triton X-100 containing a protease inhibitor cocktail (Roche)]. Lysates were incubated on ice for 10 min prior to centrifugation at 13,000 ***g*** for 10 min at 4°C. The resulting supernatant was incubated for 1.5 h with the indicated GST fusion protein (0.25 nmol of protein per reaction, unless otherwise indicated) bound to 20 μl glutathione–Sepharose beads or with 15 μl of prewashed GFP-Trap beads (ChromoTek). 50 μl of supernatant was retained for analysis of cell lysate. 10 μl 6× loading buffer was added. Beads were washed four times and boiled in 60 μl SDS-loading buffer. 20 μl samples were separated on SDS-PAGE gels, transferred onto PVDF membrane, blocked in 5% milk in TBS-T (20 mM Tris-HCl pH 7.5, 0.25 M NaCl and 0.1% Tween-20), and probed with the indicated primary antibodies followed by detection with horseradish peroxidase (HRP)-conjugated secondary antibodies. Blots were developed with an ECL kit (Biorad) and chemiluminescent signal detected and quantified using a Bio-Rad XR system and ImageLab software. Antibodies used for western blotting were: anti-GFP, Roche, 11814460001, 1:1000; anti-His_6_, Millipore, 71840, 1:1000; and anti-HA, Sigma, HA-7, 1:1000 antibodies.

### Direct binding experiments

GST, and GST–KHC^815-955^ or its variants (0.5 nmol of protein per reaction) bound to glutathione–Sepharose beads was incubated with 300 μl of 1.2 μM SKIP(1-310) or its variants for 2 h at 4°C. Beads were washed four times with 1 ml assay buffer (25 mM HEPES, pH 7.5, 150 mM NaCl, 5 mM β-mercaptoethanol) or assay buffer containing 20 mM imidazole when using His_6_-tagged proteins. Beads were resuspended in 50 μl of buffer and 10 μl of 6× SDS-loading buffer before boiling; 20 μl of each sample were loaded, and the SDS-PAGE gel was stained with InstantBlue Protein Stain (Expedeon) or analysed by western blotting as described above.

### Immunofluorescence

HeLa cells were plated at a density of 1×10^5^ per well on fibronectin-coated glass coverslips in a six-well plate 6 h prior to transfection. Cells were transfected with 0.8 μg SKIP DNA (CB6 expression vectors) using Effectene transfection reagent (Qiagen). The cells were then incubated at 37°C for 16 h. Cells were fixed at −20°C with 100% methanol for 10 min. This was followed by blocking with 1% BSA in PBS for 20 min. Cells were washed three times with PBS. Cells were then probed with the appropriate antibody (LAMP1, Cell Signaling, D2D11, 1:200 or anti-Myc, Sigma, 9E10, 1:400) diluted in blocking solution (1% BSA in PBS). After 2 h at room temperature, cells were washed three times for 5 min each time with PBS followed by incubation with a secondary fluorescent-protein-conjugated antibody (in blocking solution) for 30 min. Coverslips were washed three more times with PBS, placed cell side down in Fluor save reagent (Calbiochem). Widefield fluorescence images were collected using a Zeiss Olympus IX-81 microscope with a 40× objective running Metamorph software. Confocal images were collected using a Nikon A1 system with a 100× objective running NIS Elements.

### Quantification of lysosome distribution

To quantify lysosome distribution, widefield images were acquired at a 40× magnification. The cell perimeter was defined by thresholding equivalent saturated images and the area was scaled at 10% decrements using ImageJ. The image was binarized and the cumulative LAMP-1 distribution (relative to the whole cell) was then plotted for increasing incremental deciles (see Starling et al., 2016 for further details). Data points are from a minimum of 45 cells in three replicates and are representative of at least three independent experiments. For analysis of the resulting data, the non-linear regression function in Graphpad Prism was used to fit a centered sixth order polynominal. To compare models and assess the statistical significance of differences in distribution profiles, the extra sum of F-squares test was applied. *P* values for particular comparisons are indicated on the graphs.

### Fluorescence polarization

N-terminal carboxytetramethylrhodamine (TAMRA)-conjugated peptide used for fluorescence polarization measurements (TAMRA-SKIPWDWE; STNLEWDDSAIAPSSEDYDFGDVFPAVPSVPSTDWEDGDL supplied by BioSynthesis, Lewisville, TX). Measurements were performed on a Horiba Fluoromax-4 spectrofluorometer as described previously (Yip et al., 2016).

### Cellular kinesin-1 and MT pelleting assay

To determine the capacity of SKIP promote the association of the kinesin-1 tetramer with MTs, 0.5×10^6^ HeLa cells were plated onto a 10 cm dish and co-transfected the next day with HA–KHC and HA–KLC as well as a GFP, or GFP–SKIP(1-310) (WT, WD/AA, WE/AA, EDY/AAA or DFG/AAA variants). Cells were lysed at 24 h post transfection in 3 ml of MT stabilization buffer [100 mM PIPES pH 6.9, 5 mM MgCl_2_, 1 mM EGTA, 30% (v/v) glycerol, 0.1% (v/v) Nonidet P40, 0.1% (v/v) Triton X-100, 0.1% (v/v) Tween-20, 0.1% (v/v) β-mercaptoethanol, 100 μM GTP and 20 μM taxol] supplemented with protease inhibitors. 1 ml of lysate was subjected to ultracentrifugation to pellet intact MTs (100,000 ***g*** for 30 min at 37°C). Supernatant containing the soluble tubulin fraction was removed into a microfuge tube with 5× SDS sample buffer; 5× sample buffer was also added to the pellet fraction followed by dilution with 1 ml of MT stabilization buffer. 20 μl of each fraction were separated by SDS-PAGE electrophoresis, immunoblotted and subjected to densitometric quantitation. Data presented are the mean of three independent experiments.
